# Breeding biology of two wagtail subspecies on Ulleung Island, Korea: Amur Wagtails, *Motacilla alba leucopsis* and Black-backed Wagtails, *M. a. lugens*

**DOI:** 10.1080/19768354.2018.1496949

**Published:** 2018-07-18

**Authors:** Ji-Young Lee, Jin-Young Park, Incheol Kim, Woo-Yuel Kim, Ha-Cheol Sung

**Affiliations:** a School of Biological Sciences and Biotechnology, Chonnam National University, Gwangju, Korea; b National Institute of Environmental Research, Incheon, Korea; c Division of Ecological Survey Research, National Institute of Ecology, Seocheon-gun, Korea; d Department of Biological Sciences, College of Natural Sciences, Chonnam National University, Gwangju, Korea

**Keywords:** Breeding pairs, hybridization, reproductive success, taxonomic status, white wagtail complex

## Abstract

There is much controversy over the species and subspecies status of the white wagtail complex, which is further compounded by interbreeding between two subspecies, the Amur Wagtail (*Motacilla alba leucopsis*) and the Black-backed Wagtail (*M. a. lugens*). This study presents preliminary information on the breeding biology of both subspecies on Ulleung Island, Korea, over two breeding seasons (2012–2013). Mixed pairs of the two subspecies were common on this island, with almost 50% of all pairs being heterotypic or intermediate pairs; however, assortative mating was still present. Females of both subspecies were more likely to be paired with Amur wagtail males, whereas intermediate females were more likely to be paired with Black-backed Wagtail males. Clutch size, egg size and mass, and reproductive parameters (such as hatching success and nest success) did not significantly differ from each other. However, the mean values were low in intermediate pairs. Our results indicate no reproductive barrier between the two subspecies, but that some post-isolating mechanisms are still in progress.

## Introduction

The white wagtail (*Motacilla alba*) has a widespread breeding distribution, expanding across most of Eurasia, including western Alaska and southeastern Greenland. Over this range, nine to 13 subspecies have been recognized, based on extensive geographic variation in morphological and genetic characteristics (Cramp 1988; Snow and Perrins 1998; Alström et al. 2003; Pavlova et al. 2005). Variation exists in the color of the back (Vaurie 1959) and the head plumage patterns of breeding males (Cramp 1988), as well as in the genetic relationships among different subspecies (Pavlova et al. 2005).

To date, four subspecies of *M. alba* groups in East Asia have been recognized based on morphological and genetic characteristics (Alström et al. 2003; Gill and Donsker 2017). Two of these subspecies, *M. a. leucopsis* (Amur Wagtail) and *M. a. lugens* (Black-backed Wagtail), were initially classified as the same group members (Vaurie 1959) and then classified into different groups (Cramp 1988; Sangster et al. 1999; Pavlova et al. 2005). Furthermore, based on the genetic relationships of mitochondrial and/or nuclear DNA sequences, these two subspecies have been, again, classified as the same group (Pavlova et al. 2005) and classified as different group (Ödeen and Alström 2001; Alström et al. 2003). These results show lack of congruence with respect to the morphological and genetic patterns for the taxonomic status of the two subspecies.

Based on the morphological characteristics, one or two races are suggested to be considered as distinct species, in particular, the Black-backed Wagtail (Sætre et al. 1999; Stepanyan 2003). The Black-backed Wagtail was once regarded as a separate species from the white wagtail complex, mainly because of limited hybridization in Kamchatka and southern Ussuriland (Nazarenko 1968; Stepanyan 1978, 1983; AOU 1983, 1998). Nazarenko (1968) found that hybrids of Amur Wagtails × Black-backed Wagtails were rare, and less viable than pure parental pairs, and that there were differences between the subspecies in the timing of breeding and habitat used for breeding. However, hybrids of Amur Wagtails × Black-backed Wagtails have begun to be observed in some places of southern Japan, due to the breeding range expansion of the Black-backed Wagtails (Okayama 1984). With these hybrids, other molecular data has led to the Black-backed Wagtail back again being officially grouped with the white wagtail by the American Ornithologists’ Union (Banks et al. 2005). While this phenomenon is not an issue at the species level, it makes the subspecies status unclear. Alström et al. (2003) showed the Amur Wagtail on a DNA tree as belonging to different branches or clades based on adult male plumage, and suggested the need to clarify the relationship of the Amur wagtail with other subspecies.

In Korea, Amur Wagtails are common summer breeders, whereas Black-backed Wagtails are winter visitors (Lee et al. 2000). In particular, based on the National Natural Environment Survey by the Ministry of Environment in 2001, only six to 10 Amur Wagtails were observed in July 2001, and just two to five Black-backed Wagtails were observed in October 2001, on Ulleung Island, Gyeongbuk Province, in the East Sea of Korea (Kim and Nam 2001). Recently, a study reported more than three interbreeding pairs of these two subspecies on the island (Park et al. 2011). However, there is no information on whether the two taxa produce viable and fertile offspring, or about their pairing patterns.

Thus, to elucidate the breeding biology of Amur Wagtails and Black-backed Wagtails, we examined the proportions of breeding pairs and their reproductive performance on Ulleung Island. Specifically, we investigated the timing of breeding, pair composition, nest site selection, clutch size, length of incubation, nestling periods, and breeding success, and attempted to reveal the current taxonomic status as well as relationship between subspecies. By estimating the level of hybridization between these two subspecies, we expect to provide baseline information on the taxonomic status of the two subspecies, as well as the process of species formation.

## Materials and methods

### Study area and species

**Figure 1.**
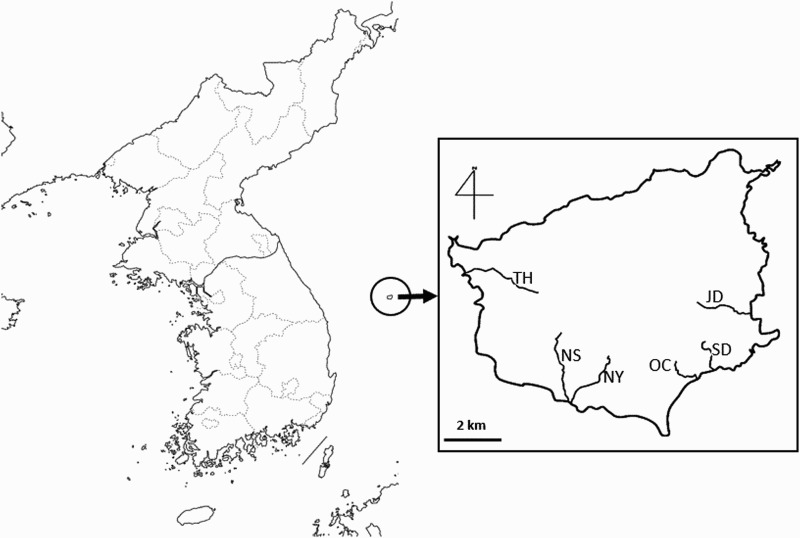
Location of Ulleung Island (encircled) on the Korean Peninsula and the six main breeding sites following streams, which were studied in 2012–2013. TH; Teaha stream (4 pairs), NS; Namseo stream (3 pairs), NY; Namyang stream (1 pair), SD; Sadong stream (3 pairs), OC: Oc stream (4 pairs), and JD; Judong stream (4 pairs).

The study was conducted on Ulleung Island (37°30′N, 131°52′E), which is located 120 km east of the Korean Peninsula (Figure 1). The island is 12 km long, 9–10 km wide, and has an area of 72.9 km^2^. The climate of this island is classified as ‘humid subtropical climate’ with an average annual temperature of 12.4°C and precipitation of 1383.4 mm (1981–2010; Korea Meteorological Administration 2011). Surveys of nests were mainly made along shoreline roads and along six streams on the west (TH; Teaha stream), southwest (NS; Namseo stream, NY; Namyang stream), southeast (SD; Sadong stream, OC: Oc stream), and east (JD; Judong stream) sides of the island.

**Figure 2.**
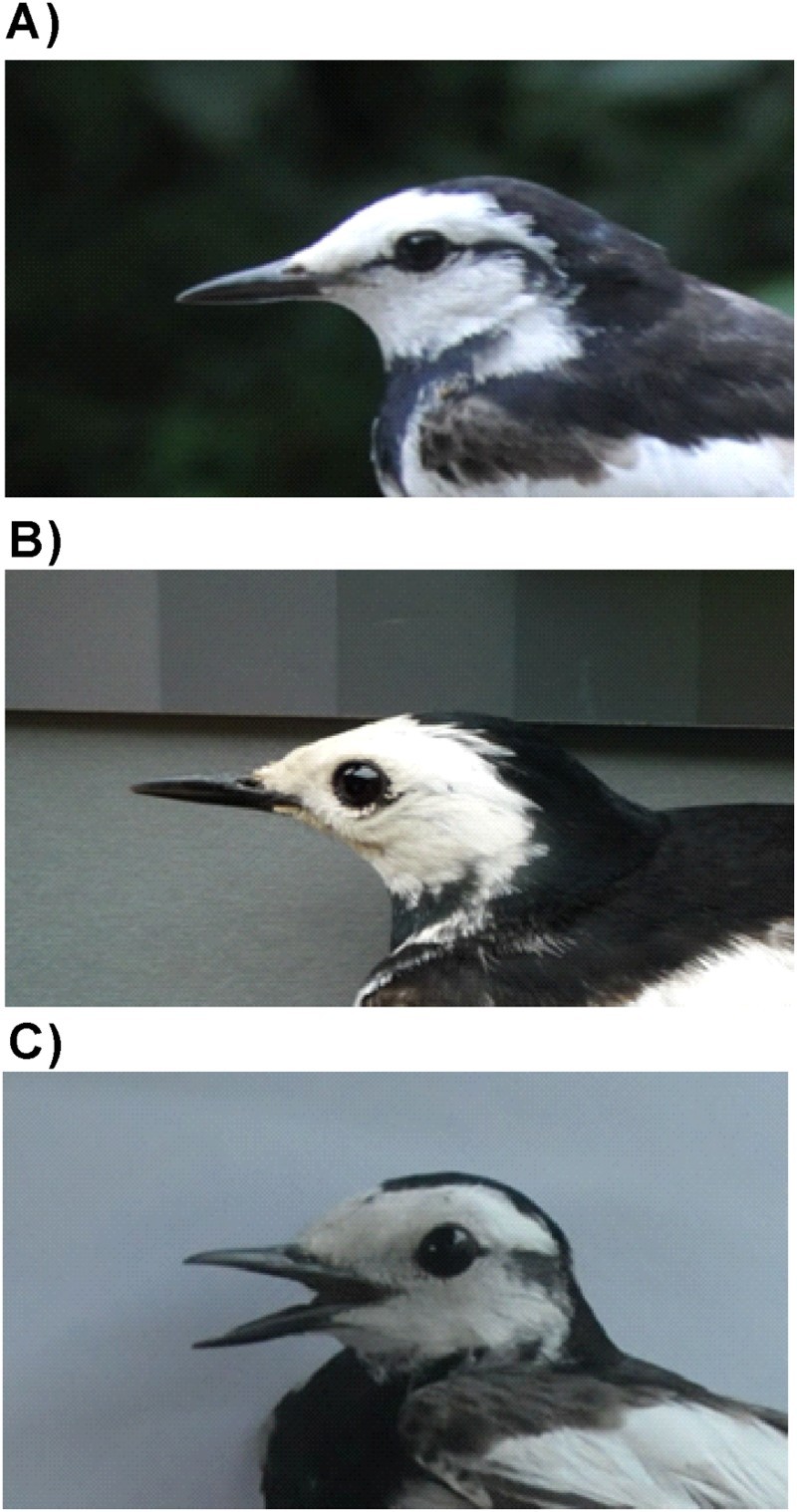
Breeding Wagtails in Ulleung Island (A) Black-backed Wagtail male, (B) Amur Wagtail male, (C) intermediate female.

The sex of both subspecies was determined by the darkness of the back, whereby females have a much darker gray back than males. Adult Amur Wagtails were easily identified from adult Black-backed Wagtails by the absence of a dark stripe through the eye (Lee et al. 2000). In addition, adult Amur Wagtails have a pure white chin and throat, with black primary coverts and alula. In comparison, adult Black-backed Wagtails have eye-stripes and some white on the primary coverts and alula. We regarded intermediate phenotypes of eye-stripes and plumages as potential hybrids, such as when the eye-stripes did not show the full characteristics of one subspecies or the other (Figure 2).

### Data collection

Data were collected during the breeding season, from early April to early July, in 2012–2013. We monitored the nest success and breeding behavior of 33 breeding pairs with 37 nests, including four second nests during both study years. Photographs were taken of all pairs. Some individuals were also caught with mist and bow nets. We then banded captured birds with a standard aluminum band provided by the Korean National Institute of Biological Resources. Each bird was given a unique combination of one or two color bands for individual recognition. To find nests, we followed nest-building females, off-duty (foraging) females from incubation, and food delivering parents. The nests were checked every second day to determine clutch size, incubation period, nest fate, and changes to habitat components. We recorded the nest location, nest size (outer minimum width, maximum length, and height of all nests), egg weight (g), egg length, and width with a caliper to the nearest 0.1 mm. The initiation of egg-laying was determined by back-dating one day per egg from the clutch completion date, and by back-dating 15 days from the dates of egg-hatching.

To estimate reproductive success, we divided the breeding pairs into three: monotypic (consisting of a single subspecies), heterotypic (consisting of mixed subspecies), and intermediate pairs (consisting of at least one hybrid), and used the data from both years because there were no differences in clutch sizes (Mann–Whitney U test: *U* = 136, *N1* = 14, *N2* = 23, *p* > 0.05) and the breeding cycles were similar between the two years. We used egg hatching success (the proportion of eggs that hatched), fledging success (the proportion of hatched chicks that fledged), and nest success (the proportion of all clutches with eggs that fledged at least one chick). Observations started in a randomly chosen order of nests, from early in the morning (sunrise) to 12:00 and for 5 h before sunset. For observations, 10 × binoculars (Nikon, Monarch) and a 20–60 × spotting scope (Swarovski, ATS-80) were used.

### Data analysis

We tested the data for significant deviations from normality before using parametric statistical tests (One-sample Kolmogorov–Smirnov test, *p* < 0.05). If the data did not meet the assumption of normality, then we used non-parametric tests. Kruskall-Wallis tests were performed to find differences among the three groups for clutch size, while one-way ANOVAs were used to detect differences in egg size and mass. To detect patterns in mating frequency observed in the pairing events between the two subspecies, we performed a cross tabulation analysis and Chi-square test. Data were analyzed using SPSS Statistics software package (v. 21, IBM Corporation). *α* = 0.05 was used to test the significance of the results. Numerical data are presented as mean ± SD.

## Results

### Pair formation

We documented a total of 33 breeding pairs of wagtails (12 pairs in 2012; 21 pairs in 2013) over the two survey years, of which we captured and banded 25 individuals (11 individuals in 2012; 14 individuals in 2013). We classified the birds based on their morphological characteristics (Table 1). Most breeding individuals (67%) on the island were Amur Wagtails, while 26% were Black-backed Wagtails and 7% were intermediates (*n* = 66). Almost half of the pairs (52%) were monotypic (16 Amur Wagtails, 1 Black-backed Wagtail), while 11 pairs (33.3%) were heterotypic and five pairs (15.2%) were intermediate. Of note, the intermediates were all females that mated with a single male Amur wagtail and four male Black-backed Wagtails. There was a marginally significant difference in proportions of females that paired with different types of males (*X*^2^ = 6.014, *df* = 2, *p* = 0.049): the females of both subspecies were more likely to pair with Amur wagtail males, while intermediate females were more likely to pair with Black-backed Wagtail males.

**Table 1.** Cross-tabulation for the frequencies of pairing events of wagtails in 2012–2013.

	Female of pair			Total
Male of pair	AF	BF	HF	
AM	16	5	1	22
BM	6	1	4	11
Total	22	6	5	33

AM; Amur wagtail male (*Motacilla alba leucopsis*), BM; Black-backed Wagtail male (*Motacilla alba lugens*), AF; Amur Wagtail female, BF; Black-backed Wagtail female, HF; intermediate female.

### Nests and eggs

**Figure 3.**
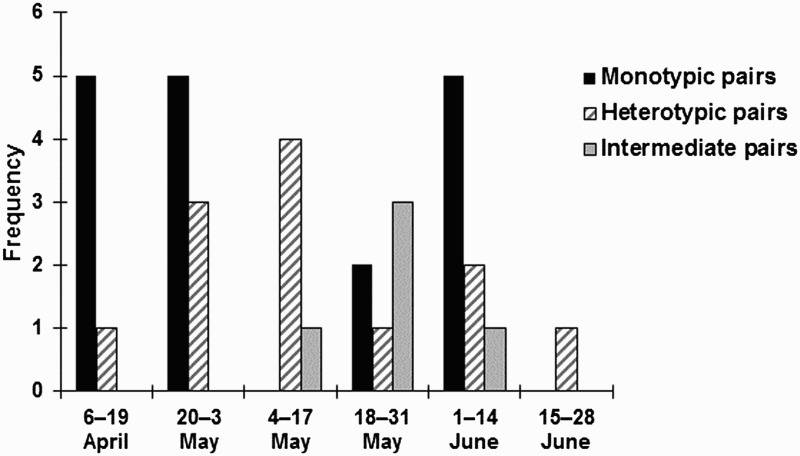
Frequency of Wagtail pairs according to clutch initiation by monotypic, heterotypic, and intermediate pairs on Ulleung Island in 2012–2013.

We located 14 nests of 12 pairs in 2012 and 23 nests of 21 pairs in 2013, all of which included two second clutches. Nests were initiated in April, with the first eggs being laid on April 7 and April 9 in 2012 and 2013, respectively. The last eggs were laid on May 26 and June 15 in 2012 and 2013, respectively. Second clutches were initiated on June 8 and May 24 in 2012 and 2013, respectively. There were clear differences in the timing of clutch initiation among the three groups of pairs (monotypic, and heterotypic, and intermediate). More monotypic pairs started laying eggs earlier than heterotypic (*n* = 11, mean 4.2 days later) and intermediate (*n* = 5, mean 15.2 days later) pairs during the two breeding seasons. In particular, intermediate pairs first bred after mid-May (Figure 3).

The most common clutch size was five eggs (4.76 ± 1.05, range 2–6, *n* = 37). The mean clutch size of intermediate pairs was lower than that of the other groups, but there were no significant differences among the three groups (Kruskall-Wallis test: *X*^2 ^= 0.079, *df *= 2, *p* > 0.05, Table 2). Egg width, length and mass were measured from 62 eggs in 12 clutches in both years (Table 3). There were no significant differences among the three groups for these three characteristics. However, the means of all three measurements were lowest for the eggs of intermediate pairs.

**Table 2.** Comparison of breeding parameters for pure Amur wagtail (*Motacilla alba leucopsis*) and Black-backed Wagtail (*Motacilla alba lugens*) pairs (monotypic), mixed pairs (heterotypic), and hybrid pairs (intermediate) in 2012–2013.

Pair	Nest no.	Clutch size	No. of eggs hatched	Hatching success (%)	No. of Fledglings	Fledging success (%)	Nest success (%)
Monotypic	20	4.7	4.4	94.6	3.9	88.6	90.0
Heterotypic	12	4.8	4.1	84.5	4.1	100.0	83.3
Intermediate	5	4.6	3.6	78.3	3.6	100.0	80.0
Total	37	4.8	4.2	89.1	3.98	93.5	86.5

**Table 3.** Comparison of egg size and mass for pure Amur wagtail (*Motacilla alba leucopsis*) and Black-backed Wagtail (*Motacilla alba lugens*) pairs (monotypic), mixed pairs (heterotypic), and hybrid pairs (intermediate) in 2012–2013.

Pair	No. egg (nest)	Width (mm)	Length (mm)	Weight (g)
Monotypic	30 (6)	15.14 ± 0.44	20.22 ± 0.71	2.36 ± 0.23
Heterotypic	21 (4)	15.24 ± 0.92	20.65 ± 0.81	2.46 ± 0.23
Intermediate	11 (2)	14.99 ± 0.32	20.08 ± 0.46	2.31 ± 0.20
Total	62 (12)	15.15 ± 0.62	20.34 ± 0.74	2.38 ± 0.23

Only females built nests and incubated eggs, while males defended territories and fed females during the breeding period. Nests were mainly located in the retaining walls along the streams (59.4%; 22/37), and a few nests were located on coastline rock cliffs (8.1%; 3/37), stone wall and clay wall located near village (18.9%; 7/37), holes or crevices in tree, stones, or under the ground (13.5%; 5/37; Table 4). The average height of the 37 nests above ground level was 2.8 m (±1.6 m) and the average distance to the nearest water line from the nests was 29.8 m (±39.8 m). The overall dimensions of the nests (*n* = 27) were measured: outside nest widths and lengths averaged 12.5 cm (±2.7) × 16.8 cm (±6.7); and the heights were 16.5 cm (±7.0). Both sexes fed nestlings. The mean duration of the incubation period between laying the first egg and hatching was 14.7 (±1.10) days (range 13–17, *n* = 25). Nestlings fledged 14–15 days after hatching (14.9 ± 0.35, *n* = 8).

**Table 4.** Nest location and breeding success of wagtails on Ulleung Island, Korea, in 2012–2013.

Nest location	2012	2013	Total	Breeding success (%)
Stream retaining wall	9	13	22	81.8
Coastline rock cliffs	0	3	3	100
Village stone or clay wall	3	4	7	85.7
Holes or crevices in trees or stones	1	2	3	100
Total	14	23	37	86.5

### Reproductive performance

Overall, the means of hatching success, fledging success, and nest success ranged from 86.5% to 93.5% (Table 2). Intermediate pairs had the lowest percentages for all measurements (clutch size, mean number of eggs hatched, and fledglings per nest, hatching and nest success), except fledging success, while monotypic pairs had the highest percentages for hatching and nest success. Predation was the main cause of nest failures. Fourteen eggs and six chicks from four nests were preyed upon over the two years. One monotypic pairs and one intermediate pair lost one nest each, while heterotypic pairs lost two nests. Five eggs from one nest of a monotypic pair were abandoned and four chicks from three nests of monotypic pairs were found dead in the nests.

## Discussion

### Wagtail breeding biology on Ulleung Island

Overall, the breeding phenology of Amur Wagtails and Black-backed Wagtails on Ulleung Island differed to that of other birds in the coastal area of southern Ussuriland, Russia, and inland Honshu, Japan, areas. Egg-laying on this island was almost one month earlier than that documented in southern Ussuriland and similar to (or slightly earlier than) Honshu. For instance, in both years, the pure Amur wagtail pairs and heterotypic pairs laid eggs on April 7–9 and April 10–25, respectively, on Ulleung Island (Figure 3). In comparison, for Black-backed Wagtails first laid eggs on May 8 in southern Ussuriland (Panov 1973) and April 27 on Honshu (Higuchi and Hirano 1983; Nakamura et al. 1984), and on May 22 for the heterotypic pairs in southern Ussuriland (Nazarenko 1968).

The early egg laying dates on Ulleung Island might influence larger clutch size (Hendricks 1997). The mean clutch size was 4.8 eggs (95% CI = 4.4–5.1) from 37 nests on this island, which was larger than that in southern Ussuriland (4 eggs; Panov 1973) and south Kuril Island, Russia, for Black-backed Wagtails (4.3 eggs; Nechaev 1969). In addition, mean egg size and mass on this island were lower than that observed in the other areas. For instance, these parameters were 15.2 (95% CI = 15.0–15.3) × 20.3 mm (95% CI = 20.2–20.5) with 2.38 g (95% CI = 2.33–2.44) for all 62 eggs of 12 nests on Ulleung Island (Table 3), but were 16.4 × 22.8with 3.28 g (5 eggs) on Honshu (Nakamura et al. 1984) and 15.9 × 21.5 (20 eggs) with 2.92 g on north Kuril Island for Black-backed Wagtails (Dement’ev and Gladkov 1954).

As for reproductive success, nest success (86.5%) on Ulleung Island was higher than that on Honshu, which documented 20% (1/5) and 70.6% (12/17) at two sites over a three-year period (Higuchi and Hirano 1983; Nakamura et al. 1984). These values were even lower than those obtained for the intermediate pairs (80.0%) on Ulleung Island. Hatching success and fledging success of White Wagtails in Central Finland were 80.9% and 77.0% respectively, which were also lower than those in this island (Leinonen 1973). The main cause of nest failures was predation on Ulleung Island (10.8%, 4/37), but it did not severely affect nest success when compared with the success of inland birds. The island can provide isolation from avian nest predators (i.e. snakes and mammals), reducing the risk of predation (Fontaine and Martin 2006; Ocampo and Londoňo 2015). In addition, human beings as well as predation were main causes of nest failure in a population of Central Finland (Leinonen 1973). Thus, our results on the early egg-laying dates, larger clutch size, and higher nest success with low predation risk indicate the benefits to wagtails of breeding on the isolated island of Ulleung compared with other areas.

### Wagtail hybridization and fitness

Our data demonstrate Black-backed Wagtails suffer more from hybridization than Amur Wagtails. The latter species was more abundant than the former species on Ulleung Island. Furthermore, there were more pure pairs of Amur Wagtails than Black-backed Wagtails. Black-backed Wagtails tended to pair more frequently with intermediate females. Similarly, another study showed that the rarer pied flycatcher (*Ficedula hypoleuca*) was also more involved in hybridization than the more common collared flycatcher (*F. albicollis*, Sætre et al. 1999; Veen et al. 2001). The authors suggested that this pattern reflected the relative frequency of available individuals when they search for mates with time constraints (Real 1990). However, for the two subspecies of Xantus’s Murrelets (*Synthliborahus hypoleucus scrippsi,* and *S. h. hypoleucus*), there were no differences in the timing of breeding among monotypic, heterotypic, and intermediate pairs. Furthermore, there was no clear evidence of the rarer subspecies (*S. h. hypoleucus)* being more involved in heterotypic pairs than the more common species (*S. h. scrippsi*, Wolf et al. 2005).

The timing of breeding might play a role in the level of hybridization detected in this study. The observed males that first arrived on Ulleung Island were mainly the Amur Wagtails, with the Black-backed Wagtails arriving as the breeding seasons progressed. The first clutch of a pure Amur wagtail pair was 21 days earlier than that of a pure Black-backed Wagtail pair in 2012. There was no Black-backed Wagtail pair in 2013. The first egg-laying dates of heterotypic pairs occurred three day later compared to monotypic pairs in 2012 (with an average 4.2 day gap in both years). The clutch initiation pattern of the breeding pairs indicates that mixed pairing occurs whenever both species are available at the time of mating, and that late attempts to mate by late arriving Black-backed Wagtails tend to be hybridized. In addition, the presence of a high proportion of heterotypic or intermediate pairs and their viable offspring suggest no reproductive barrier exists between the two subspecies. In particular, the lower successful reproductive performance of the intermediate pairs also suggests selection against hybrids is progressing. Breeding pairs with intermediate males were not observed in either study year, while intermediate females paired much later than other females. Thus, there might be issues with the courtship behavior of males (Mayr 1970; Alatalo et al. 1982).

When comparing reproductive performance the clutch size, and egg size and mass of intermediate pairs were smaller on average than those of monotypic and heterotypic pairs (Table 3). In addition, both hatching success and nest success were lowest in intermediate pairs. Although not statistically significant, the observed differences in reproductive performance might imply that intermediate pairs had lower fitness than either monotypic or heterotypic pairs. Similarly, less fit hybrids were reported between two flycatcher species, the Pied Flycatcher (*Ficedula hypoleuca*) and Collared Flycatcher (*F. albicollis*). In this study fewer flycatcher eggs hatched in pairs involving a hybrid mate than in pure or heterotypic pairs. In particular, pairs with a female hybrid were more likely to have infertile eggs than pairs with a male hybrid (Sætre et al. 1999). The gender specific tendency to be sterile is known as Haldane’s rule (Haldane 1922), with females being the heterogametic sex in the current study. However, it was not possible to assess this phenomenon in this study, due to the lack of pairs with a male hybrid. For two subspecies of Xantus’s Murrelets, pairs with a hybrid mate showed mixed results with respect to hatching success (100% [1/1] for two intermediate pairs and 25% [1/4] for one member of a pair). Thus, more data are needed to obtain objective information on hybrid fitness. Our results again indicate the presence of a potential isolating mechanism. However, unlike our results, a longitudinal study of Darwin’s finches (*Geospiza* spp.) showed fitness was higher in hybrids than in pure species (Grant and Grant 1992).

### Taxonomic status

Extensive variation in the White Wagtail complex has received the focus of many ornithologists regarding the taxonomic concept (Phillimore and Owens 2006; Semenov et al. 2010; Semenov et al. 2017). Even though there remains controversy on the taxonomic status of the species, because of the unclear identification of distinct phylogenetics from the other subspecies complex (Pavlova et al. 2005), the wagtails of the two subspecies on Ulleung Island might be located within the same species level as subspecies. This is because: (1) interbreeding between the two subspecies is common (about 50% of heterotypic and intermediate pairs); (2) hybrids are viable and fertile; and (3) geographical distribution barriers remain between the two subspecies (Alström et al. 2003). However, positive assortative mating exists, with generally lower reproductive success in intermediate pairs. These results imply that some post isolating mechanisms are in progress. In conclusion, additional long-term studies on the ecology, behavior, and molecular systematics of wagtails are required to confirm this relationship.

## References

[CIT0001] Alatalo RV, Gustafsson L, Lundberg A. 1982 Hybridization and breeding success of the collared and the pied flycatcher on the Island of Gotland. Auk. 99:285–291.

[CIT0002] Alström P, Mild K, Zetterström D. 2003 Pipits and Wagtails of Europe, Asia and North America. Identification and systematics. London: Christopher Helm.

[CIT0003] American Ornithologists’ Union 1983 Check-list of North American birds. 6th ed Washington, DC: American Ornithologists’ Union. 10.1126/science.ns-7.168.374-a 17739997

[CIT0004] American Ornithologists’ Union 1998 Check-list of North American birds. 7th ed Washington, DC: American Ornithologists’ Union. 10.1126/science.ns-7.168.374-a 17739997

[CIT0005] Banks RC, Cicero C, Dunn JL, Kratter AW, Rasmussen PC, Remsen JV, Rising JD, Stotz DF. 2005 Forty-sixth supplement of the American ornithologists’ union checklist of North American birds. Auk. 122:1026–1031. doi: 10.1642/0004-8038(2005)122[1026:FSTTAO]2.0.CO;2

[CIT0006] Cramp S. 1988 Handbook of the birds of Europe. Vol. 5, the Middle East and North Africa. Oxford: Oxford University Press.

[CIT0007] Dement’ev GP, Gladkov NA. 1954 Family Motacillidae. In: Dement’ev GP, Gladkov NA, editors. Vol. 5, birds of the Soviet Union. Moscow: Nauka; p. 591–594.

[CIT0008] Fontaine JJ, Martin TE. 2006 Parent birds assess nest predation risk and adjust their reproductive strategies. Ecol Lett. 9:428–434. doi: 10.1111/j.1461-0248.2006.00892.x 16623728

[CIT0009] Gill F, Donsker D, editors. 2017 IOC world bird list (v.7.3). doi:10.14344/IOC.ML.7.3.

[CIT0010] Grant PR, Grant BR. 1992 Hybridization of bird species. Science. 256:193–197. doi: 10.1126/science.256.5054.193 17744718

[CIT0011] Haldane JBS. 1922 Sex ratio and unisexual sterility in hybrid animals. J Genet. 12:101–109. doi: 10.1007/BF02983075

[CIT0012] Hendricks P. 1997 Geographical trends in clutch size: a range-wide relationship with laying date in American Pipits. Auk. 114:773–778. doi: 10.2307/4089300

[CIT0013] Higuchi H, Hirano T. 1983 Comparative ecology of White and Japanese Wagtails, *Motacilla alba, M. grandis*, in winter. Tori. 32:1–11.

[CIT0014] Kim CH, Nam KB. 2001 The birds of Ulleungdo and Dokdo, ecological survey report of the natural environment. Seoul: Ministry of Environment p. 307–320.

[CIT0015] Korea Meteorological Administration 2011 Climatological normal of Korea (30 years) in Ulleungdo. Seoul: Korea Meteorological Administration.

[CIT0016] Lee WS, Ku TH, Park JY. 2000 A field guide to the birds of Korea. Seoul, Korea: LG Evergreen Foundation.

[CIT0017] Leinonen M. 1973 On the breeding biology of the White Wagtail *Motacilla alba* in Central Finland. Ornis Fenn. 50:53–82.

[CIT0018] Mayr E. 1970 Populations, species, and evolution. Cambridge, MA: Belknap Press.

[CIT0019] Nakamura S, Hashimoto H, Sootome O. 1984 Breeding ecology of *Motacilla alba* and *M. grandis* and their interspecific relationship. J Yamashina Inst Ornith. 16:114–135. doi: 10.3312/jyio1952.16.114

[CIT0020] Nazarenko AA. 1968 On the character of interrelations of two forms of Pied Wagtails in south Ussuriland. Problemi Evolutsii. 1:195–201. Russian with English summary.

[CIT0021] Nechaev VA. 1969 The birds of Southern Kuril Islands. Leningrad: Nauka.

[CIT0022] Ocampo D, Londoňo GA. 2015 Tropical montane birds have increased nesting success on small river islands. Auk. 132:1–10. doi: 10.1642/AUK-14-71.1

[CIT0023] Ödeen A, Alström P. 2001 Evolution of secondary traits in wagtails (genus *Motacilla*). In: Odeen A, editor. Effects of post-glacial range expansion and population bottlenecks on species richness [Ph.D. thesis]. Uppsala: Uppsala University; p. 1–42.

[CIT0024] Okayama H. 1984 Interbreeding between different subspecies of the White Wagtail *Motacilla alba*. Tori To Shizen. 35:1–4. Japanese.

[CIT0025] Panov EN. 1973 The birds of South Ussuriland. Novosibirsk: Nauka.

[CIT0026] Park JY, Kwon YS, Choi YS, Seo JH, Kim MR. 2011 First breeding record of the Black-backed Wagtail *Motacilla alba lugens* in Korea. Paper presented at: 2011 annual conference of the ornithological society of Korea; April 29–May 1; Shinan, Korea.

[CIT0027] Pavlova A, Zink RM, Rohwer S, Koblik EA, Red’kin YA, Fadeev IV, Nesterov EV. 2005 Mitochondrial DNA and plumage evolution in white wagtails. J Avian Biol. 36:322–336. doi: 10.1111/j.0908-8857.2005.03373.x

[CIT0028] Phillimore AB, Owens IPF. 2006 Are subspecies useful in evolutionary and conservation biology? Proc R Soc Lond B. 273:1049–1053. doi: 10.1098/rspb.2005.3425 PMC1560251 16600880

[CIT0029] Real LA. 1990 Search theory and mate choice. I. Models for single-sex discrimination. Am Nat. 136:376–405. doi: 10.1086/285103

[CIT0030] Sangster G, van den Berg AB, van Loon AJ, Roselaar CS. 1999 Dutch avifaunal list: species concepts, taxonomic instability, and taxonomic changes in 1977–1998. Ardea. 87:139–166.

[CIT0031] Sætre GP, Král M, Bureš S, Ims RA. 1999 Dynamics of a clinal hybrid zone and a comparison with island hybrid zones of flycatchers (*Ficedula hypoleuca* and *F. albicollis*). J Zool. 247:53–64. doi: 10.1111/j.1469-7998.1999.tb00192.x

[CIT0032] Semenov GA, Scordato ESC, Khaydarov DR, Smith CCR, Kane NC, Safran RJ. 2017 Effects of assortative mate choice on the genomic and morphological structure of a hybrid zone between two bird subspecies. Mol Ecol. 2017:1–15. doi:10.1111/mec.14376. 28987006

[CIT0033] Semenov G, Yurlov A, Khaydarov D. 2010 Hybridization of *Motacilla alba Linnaeus*, 1758, and *M. (a.) personata* Gould, 1861, in the south of Siberia. Contemp Probl Ecol. 3(5):579–586. doi: 10.1134/S1995425510050127

[CIT0034] Snow DW, Perrins CM, editors. 1998 The birds of the Western Palearctic concise edition. Vol. 2, passerines. Oxford: Oxford University Press.

[CIT0035] Stepanyan LS. 1978 Composition and distribution of birds of the USSR. Passeriformes. Moscow: Nauka Russian.

[CIT0036] Stepanyan LS. 1983 Superspecies and sibling species in avifauna of the USSR. Moscow: Nauka Russian.

[CIT0037] Stepanyan LS. 2003 Conspectus of the Ornithological fauna of Russia and adjacent territories (within the borders of the USSR as a historical region). Moscow: Academkniga Russian.

[CIT0038] Vaurie C. 1959 The birds of the palearctic fauna. Vol. 1, order passeriformes. London: Witherby.

[CIT0039] Veen T, Borge T, Griffith SC, Saetre GP, Bures S, Gustafsson L, Sheldon BC. 2001 Hybridization and adaptive mate choice in flycatchers. Nature. 411:45–50. doi:10.1038/35075000. 11333971

[CIT0040] Wolf S, Phillips C, Zepeda-Dominguez JA, Albores-Barajas Y, Martin P. 2005 Breeding biology of Xantus’s Murrelet at the San Benito Islands, Baja California, Mexico. Mar Ornithol. 33:123–129.

